# Gastric leak closure after the misdeployment of a lumen-apposing metal stent

**DOI:** 10.1055/a-2155-5080

**Published:** 2023-08-30

**Authors:** Giuseppe Grande, Matteo Gottin, Lorenzo Carloni, Silvia Cocca, Salvatore Russo, Rita Conigliaro, Helga Bertani

**Affiliations:** 1 Gastroenterology and Digestive Endoscopy Unit, Azienda Ospedaliero Universitaria di Modena, Modena, Italy; 2 Gastroenterology Research Unit, Department of Experimental and Clinical Biomedical Sciences “Mario Serio”, University of Florence, Florence, Italy; 3 Department of Medical and Surgical Science – DIMEC, Alma Mater Studiorum, University of Bologna, Bologna, Italy

Endoscopic ultrasound-guided cystogastrostomy (EUS-CG) represents the main therapeutic modality for treatment of pancreatic fluid collections 1 2 . While much has been written regarding technical advantages of lumen-apposing metal stents (LAMSs), there are limited data on the complications, which range from 7 % to 15 % 3 4 .

A 66-year-old woman had post-endoscopic retrograde cholangiopancreatography necrotizing pancreatitis and symptomatic pancreatic fluid collection that caused compression and dislodgment of both the stomach and duodenum. She underwent EUS-CG with placement of a 20 × 10 mm LAMS, followed by placement of a plastic double-pigtail stent inside the LAMS.

The day after the procedure, she experienced progressive epigastric pain and vomiting with abdominal tenderness. A computed tomography scan confirmed the suspicion of LAMS dislocation ( Fig. 1 ).

**Fig. 1:**
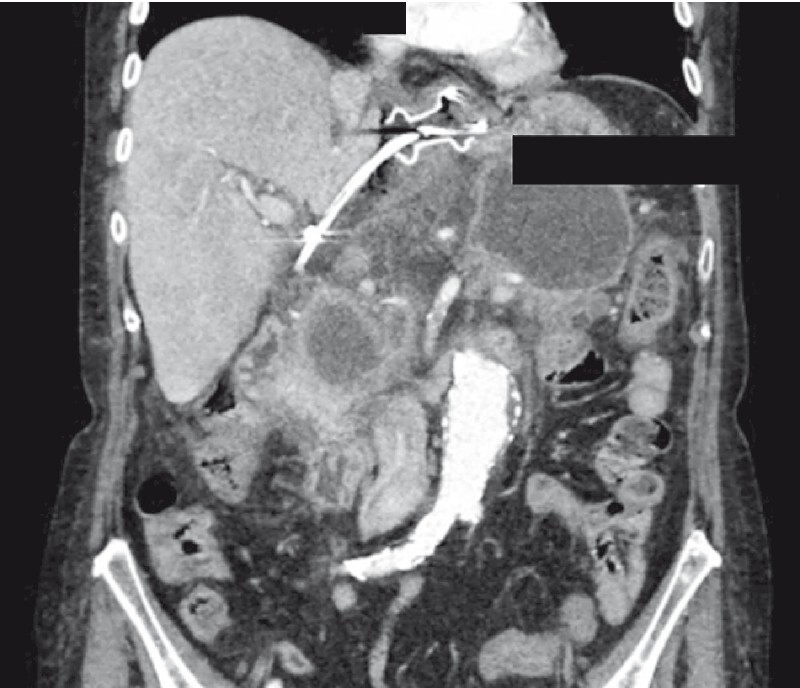
Computed tomography scan showed the lumen-apposing metal stent and the pigtail stent close to the hepatic hilus.

Once in our hospital, an upper endoscopy showed the proximal flange of the LAMS located just above the gastric cardia while the distal flange was displaced in the peritoneal space close to the hepatic hilus ( Fig. 2 ). The stents were removed, revealing a gastric parietal defect of 20–25 mm, surrounded by ulcerated tissue. The defect was immediately sutured using the OverStitch endoscopic suturing system (Apollo Endosurgery, Austin, Texas, USA) mounted on a single-channel gastroscope. Given the narrow space and the poor distensibility, four continuous running stitches were placed around the edge of the leak; however, a residual orifice of 3–4 mm was observed and an 11 mm over-the-scope clip, traumatic-type, was applied ( Video 1 ). Both endoscopic and fluoroscopic check showed no contrast extravasation outside the stomach.

**Fig. 2:**
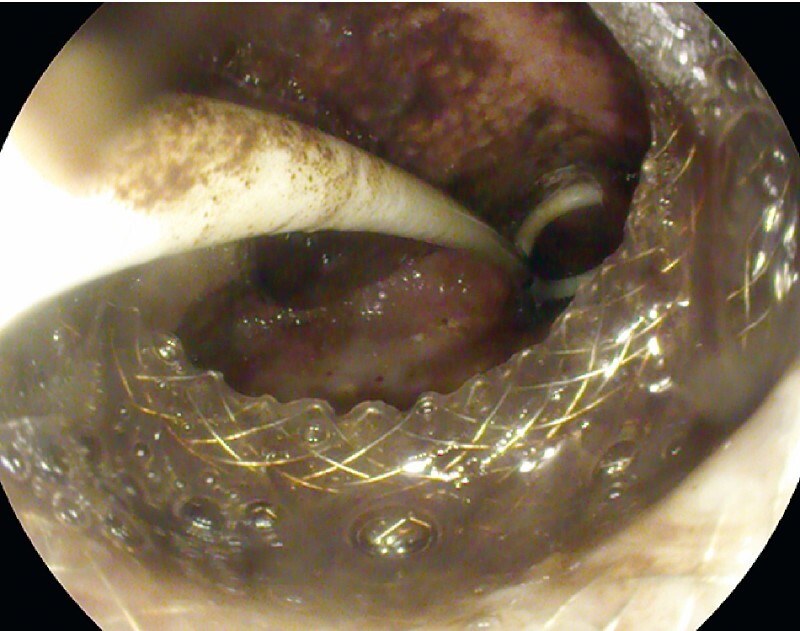
Endoscopic view, showing malposition of the lumen-apposing metal stent and pigtail stent in the abdominal cavity.

**Video 1** Gastric defect after lumen-apposing metal stent misdeployment sutured using the OverStitch system (Apollo Endosurgery, Austin, Texas, USA).


The patient showed rapid clinical improvement and a new EUS-CG was performed a few days later.

LAMS misdeployment and migration are serious adverse events 3 . In most cases the diagnosis is immediate, but sometimes the instability and malposition toward the target lesion can lead to delayed migration, resulting in continuous passage of corrosive gastric fluid into the abdomen. After stent removal, the wall defect can be difficult to treat with either through-the-scope or over-the-scope clips, especially if a 20 mm LAMS has been used 4 5 . As in the current case, the prompt application of a suturing device allows the closure, or at least the reduction of the parietal leak, avoiding further and more invasive intervention.

Endoscopy_UCTN_Code_TTT_1AO_2AI

## Notes

Endoscopy E-Videos

https://eref.thieme.de/e-videos
*E-Videos* is an open access online section of the journal *Endoscopy* , reporting on interesting cases and new techniques in gastroenterological endoscopy. All papers include a high-quality video and are published with a Creative Commons CC-BY license. Endoscopy E-Videos qualify for HINARI discounts and waivers and eligibility is automatically checked during the submission process. We grant 100% waivers to articles whose corresponding authors are based in Group A countries and 50% waivers to those who are based in Group B countries as classified by Research4Life (see: https://www.research4life.org/access/eligibility/ ). This section has its own submission website at https://mc.manuscriptcentral.com/e-videos
